# Patient characteristics, treatment patterns, and survival outcomes for patients with malignant pleural mesothelioma in Denmark between 2011 and 2018: a nationwide population-based cohort study

**DOI:** 10.2340/1651-226X.2024.34802

**Published:** 2024-08-08

**Authors:** Jens Benn Sørensen, Paul Baas, Szimonetta Komjáthiné Szépligeti, Alma B. Pedersen, Søren P. Johnsen, Robert Carroll, Minouk J. Schoemaker, Caroline Rault, Melinda J. Daumont, Vera Ehrenstein

**Affiliations:** a Department of Oncology, Rigshospitalet, Copenhagen, Denmark; b Department of Thoracic Oncology, Netherlands Cancer Institute, Amsterdam, The Netherlands; c Department of Clinical Epidemiology, Aarhus University and Aarhus University Hospital, Aarhus, Denmark; d Department of Clinical Medicine, Aarhus University, Aarhus, Denmark; e Danish Center for Health Services Research, Department for Clinical Medicine, Aalborg University, Aalborg, Denmark; f Centre for Observational Research and Data Science, Bristol Myers Squibb, Uxbridge, UK; g Real World Solutions, IQVIA, Amsterdam, The Netherlands; h Data Gnosis, Rennes, France; i Worldwide Health Economics & Outcomes Research, Bristol Myers Squibb, Braine-L’Alleud, Belgium

**Keywords:** Cohort study, Denmark, epidemiology, malignant pleural mesothelioma, registries

## Abstract

**Background:**

Malignant pleural mesothelioma (MPM) is a rare thoracic malignancy with poor prognosis and limited treatment options. Immunotherapy shows potential for improved outcomes; however, real-world evidence on its use will take time to accumulate. This study examined patient characteristics, treatment patterns, overall survival (OS), and predictors of mortality among patients diagnosed with MPM in Denmark prior to the introduction of newer treatments.

**Methods:**

This historical cohort study based on routinely collected Danish National Registry data included adults newly diagnosed with MPM between 01 January 2011 and 31 May 2018. Summary statistics were used to describe patient characteristics and initial treatment. OS was estimated using Kaplan-Meier methods; Cox regression was used to compare patient mortality against the (age/sex-matched) general population and to investigate mortality predictors.

**Results:**

Overall, 880 patients were included; 44% had advanced MPM, 37% had non-advanced MPM, and 19% had unknown MPM stage. Median age at diagnosis was 71.9 years, and 82% of the patients were male. Within 180 days of diagnosis, no treatment was recorded for 215 patients (54%) with advanced MPM and 150 (46%) with non-advanced MPM. Median time-to-initial treatment (interquartile range) was 47 days (31–111) overall, 40 days (28–77) in patients with advanced MPM, and 53 days (35–121) with non-advanced MPM. Median OS was 13.7 months overall (non-advanced MPM: 18.0 months vs. advanced MPM: 10.0 months). Predictors of higher mortality were older age at diagnosis, histology, and advanced MPM stage.

**Interpretation:**

These findings provide a baseline upon which to evaluate MPM epidemiology as newer treatments are adopted in routine practice.

## Background

Malignant pleural mesothelioma (MPM) is a rare malignancy originating in the serosal cells of the pleura [1] with around 35,000 new cases and 30,000 deaths per year globally [2]. Based on data from Denmark, the United Kingdom, France, and the Netherlands, annual incidence rates in males range from 1.9 to 3.4 new cases per 100,000 [3, 4]. The incidence of MPM has increased over the past decade due to an approximate 40-year latency period between exposure to asbestos and the development of disease [5, 6]. In 1972, asbestos was banned in Denmark for use in insulation, followed by a near-total ban in 1986 and a complete ban in 2004 [7]. Despite the widespread bans on the use of asbestos, over 80% of MPM cases are attributable to occupational exposure, as reflected in the higher proportion of affected males [4, 8, 9]; thus, it is considered a largely preventable disease [3]. It is expected that the incidence of MPM will continue to rise in low- and middle-income countries where asbestos use remains common [10].

The prognosis for patients with MPM is poor, with a median overall survival (OS) of 8–14 months from diagnosis [11, 12] and a 5-year relative survival of 12% [13]. Furthermore, available treatment options are limited [12]. Mesotheliomas are categorized into three histological subtypes: epithelioid, biphasic (mixed), and sarcomatoid [14]; epithelioid is the most common subtype, and non-epithelioid tumours are associated with a poorer prognosis after treatment (median OS, 8–13 months) [15, 16]. Until 2020, platinum-based chemotherapy (cisplatin or carboplatin) plus pemetrexed was the only systemic anticancer therapy (SACT) regimen approved for use in the treatment of MPM, providing a modest (12.1 months vs. 9.3 months in the control arm) improvement in median OS [17]; this regimen was considered the first-line standard of care for patients with non-resectable tumours (in patients with reduced renal function, single-agent chemotherapy was recommended) [3, 18]. Newer approved therapies, such as nivolumab plus ipilimumab, a combination immunotherapy, show potential for improvement in the prognosis for patients with MPM. Based on positive results from the CheckMate 743 trial [19], the nivolumab plus ipilimumab regimen was first approved by the United States Food and Drug Administration in 2020 and then by the European Medicines Agency in 2021 as the first-line treatment for adult patients with unresectable MPM [3, 20, 21]; as of September 2021, it has been included in the European Society for Medical Oncology treatment recommendations for first-line use in patients with MPM [22]. It has been approved for reimbursement in Denmark for use in patients with non-epithelioid histology since March 2022 [23].

Real-world evidence from newer MPM treatments such as immunotherapy will take time to accumulate; therefore, up-to-date data on the MPM patient population prior to the availability of these treatments could provide a baseline upon which to evaluate their benefits as they are introduced into clinical practice [24]. Comprehensive and robust population-based data sources, such as those held in the Danish national registries, are an optimal setting for such an exercise [25]. As part of the I-O Optimise multinational research initiative focused on exploring real-world management of thoracic malignancies [26], this cohort study aimed to provide information on patient characteristics, describe initial treatment patterns, estimate OS, and elicit predictors associated with mortality among patients diagnosed with MPM in Denmark.

## Materials and methods

### Study design, setting, and participants

This was a historical, nationwide population-based cohort study, using data linked from the Danish Civil Registration System [27], the Danish Cancer Registry [28], and the Danish National Registry of Patients [29]. The study included all patients identified in the Danish Cancer Registry aged ≥ 18 years with newly diagnosed MPM (International Classification of Disease, 10th Revision [ICD-10] code: C45.0) between 01 January 2011 and 31 May 2018. Patients with missing data on age or sex or with any primary malignancy within 5 years of the MPM diagnosis (except non-melanoma skin cancer; ICD-10 code: C44) were excluded. Patients were classified into three subgroups based on MPM stage and histological type at diagnosis as described in Supplementary Figure S1 [30]. Patients were included between 01 January 2011 and 31 May 2018 and followed until 31 December 2018.

### Variables

Histology was described based on the ICD-O-3 codes: 90503 for non-specified mesothelioma (including cases where pleural malignancy is present and supported by thoracic imaging, but subtyping is not possible), 90513 for sarcomatoid, 90523 for epithelioid, and 90533 for biphasic mesothelioma (defined as either predominantly epithelioid with > 10% sarcomatoid histology or predominantly sarcomatoid with > 10% epithelioid histology). Tumour, node, metastasis (TNM) staging followed the International Mesothelioma Interest Group classification system [31]. Date of death due to any cause was extracted from the Danish Civil Registration System.

### Statistical analysis

Patient characteristics (age, sex, comorbidity profile, tumour stage, and histology) were collected at diagnosis (Supplementary Table S1). Summary statistics were used to describe patient baseline characteristics and initial treatment. The index date was defined as the date of initial diagnosis for all analyses apart from the initial treatment analyses, where the index date was the date of diagnosis plus 180 days needed to ascertain the treatment category. Initial treatment was defined as the combination of different treatments the patient received within 180 days of MPM diagnosis; to examine OS by treatment category, the index date was defined as the date of diagnosis plus 180 days (Supplementary Table S2). OS was estimated using Kaplan-Meier methods [32] and was defined as time from date of diagnosis to date of death from any cause or until censoring by emigration or end of follow-up. Risk of death was computed using the 1-Kaplan-Meier estimator. Cox’s regression [33] was used to identify predictors for mortality within the overall patient population with MPM and by MPM subgroup. Mortality rates in patients with MPM were described using age-standardization with MPM-2011 population as the standard.

### Ethical aspects

Required approvals for the study were received via registration with Aarhus University (record number AU812). Ethical approval is not required in Denmark for studies based exclusively on routinely collected registry data; therefore, informed consent was not required. According to Danish legislation, counts of less than five patients are considered sensitive data and therefore must be masked when reported. In addition, rounding to the nearest 5 patients was applied whenever necessary to apply masking.

## Results

### Patient characteristics

The study population included 880 adult patients diagnosed with MPM between 01 January 2011 and 31 May 2018; 325 (37%) patients were classified as having non-advanced MPM, 390 (44%) as having advanced MPM, and 165 (19%) as having unknown MPM stage. Most (82%, *n =* 720) patients were male, with a median age of 71.9 years (interquartile range [IQR], 65.5–77.6) (Table 1). In the overall population, histology distribution was 40% (*n =* 345) biphasic, 32% (*n =* 285) epithelioid, 11% (*n =* 95) sarcomatoid, and 18% (*n =* 155) unspecified; 36% (*n =* 315) of patients were classified as TNM stage I–II, 22% (*n =* 195) as stage III, and 21% (*n =* 180) as stage IV. Around half of patients (*n =* 410) had at least one comorbidity at MPM diagnosis, per the Charlson Comorbidity Index (CCI), and the median follow-up time in the overall population was 12.7 months (IQR, 6.2–22.0) (Table 1).

**Table 1:** Clinical and demographic characteristics of patients with MPM at diagnosis, both overall and by subgroup.

	All (*N* = 880)	Non-advanced MPM (*n* = 325)	Advanced MPM (*n* = 390)	Unknown MPM (*n* = 165)
**Age, years**				
Median	71.9	70.4	72	74.3
IQR	65.5–77.6	64.6–76.2	65.6–77.9	66.9–79.1
**Male, *n* (%)**	720 (82)	260 (81)	325 (83)	135 (83)
**Histology, *n* (%)**				
Biphasic	345 (40)	150 (47)	130 (33)	70 (41)
Epithelioid	285 (32)	125 (39)	105 (27)	50 (32)
Sarcomatoid	95 (11)	0	95 (24)	0
Not specified	155 (18)	45 (15)	65 (16)	45 (27)
**Follow-up time, months**				
Median	12.7	15.9	9.5	13.9
IQR	6.2–22.0	9.3–27.8	4.2–19.0	5.9–21.8
**TNM stage, *n* (%)**				
I–II	315 (36)	280 (86)	30 (8)	0
III	195 (22)	40 (12)	155 (40)	0
IV	180 (21)	0	180 (46)	0
Unknown	190 (22)	0	25 (6)	165 (100)
**CCI, *n* (%)**				
Low (0)	470 (53)	180 (55)	205 (52)	85 (52)
Medium (1–2)	320 (36)	110 (34)	150 (38)	60 (37)
High (>2)	90 (10)	35 (11)	35 (9)	20 (11)

All characteristics were collected at time of diagnosis. All frequencies rounded to nearest 5, also affecting reported percentages.

CCI: Charlson Comorbidity Index; IQR: interquartile range; MPM: malignant pleural mesothelioma; TNM, tumour, node, metastasis.

### Treatment patterns in patients diagnosed with MPM

**Figure 1:**
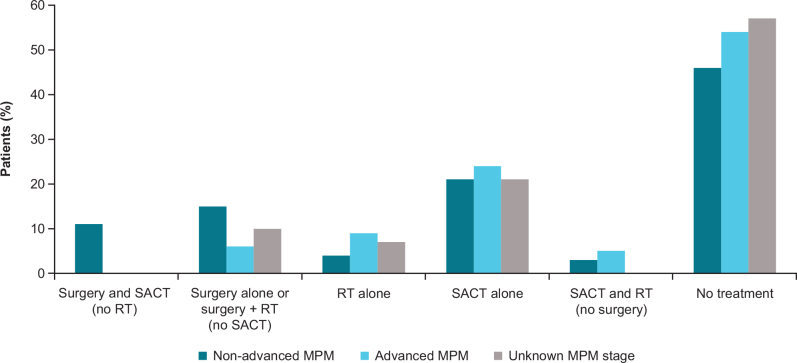
Initial therapy combination for patients with MPM. MPM: malignant pleural mesothelioma; RT: radiotherapy; SACT: systematic anticancer therapy.

Among patients with a treatment record within 180 days of MPM diagnosis, the median time-to-initial recorded treatment was 47 days (IQR, 31–111) overall, 40 days (IQR, 28–77) in patients with advanced MPM, and 53 days (IQR, 35–121) in patients with non-advanced MPM (Table 2). In the overall patient population, SACT was recorded in 31% (*n* = 275) of patients with MPM, radiotherapy (RT) was recorded in 11% (*n* = 95), and surgery (decortication of pleura with curative intent in all cases) was recorded in 15% (*n* = 135) (Table 2). In patients with non-advanced MPM, SACT alone was recorded in 21% (*n =* 65) of patients, and 46% (*n =* 150) received no treatment during the 180 days post diagnosis of MPM (Figure 1; Supplementary Table S3). In patients with advanced MPM, SACT alone was recorded in 24% (*n =* 90) of patients, and 54% (*n =* 215) had no record of treatment in the first 180 days post-MPM diagnosis (Figure 1; Supplementary Table S3). Pemetrexed alone was the most recorded chemotherapy in the advanced MPM group (17%; *n =* 65) followed by carboplatin (10%; *n =* 40) (Table 2).

**Table 2:** Initial treatment for patients with MPM.

	All (*N* = 880)	Non-advanced MPM (*n* = 325)	Advanced MPM (*n* = 390)	Unknown stage MPM (*n* = 165)
**Time from diagnosis to initial treatment, days**				
Median, IQR	47.0, 31.0–111.0	53.0, 35.0–121.0	40.0, 28.0–77.0	52.0, 33.0–125.0
**Treatment type, *n* (%)**				
Any surgery	135 (15)	85 (26)	30 (8)	20 (13)
Any RT	95 (11)	25 (8)	55 (14)	15 (9)
Any SACT	275 (31)	115 (35)	120 (30)	40 (26)
**Surgery type, *n* (%)**				
Pleurectomy/decortication of pleura	135 (15)	80 (25)	30 (8)	20 (13)
**Radiation types, *n* (%)**				
Conventional	45 (5)	10 (4)	30 (7)	5 (–)
Other^a^	95 (11)	25 (8)	55 (14)	15 (9)
**Chemotherapy, *n* (%)**				
Pemetrexed	140 (16)	50 (16)	65 (17)	20 (12)
Cisplatin + pemetrexed	95 (11)	55 (16)	35 (8)	10 (6)
Carboplatin	85 (10)	30 (9)	40 (10)	15 (9)
Carboplatin + pemetrexed	60 (7)	25 (8)	25 (6)	10 (6)
Vinorelbine	35 (4)	20 (6)	10 (3)	5 (–)
Cisplatin	20 (2)	10 (3)	5 (–)	5 (–)
Gemcitabine	0 (0)	0 (0)	0 (0)	0 (>0)

All frequencies rounded to nearest 5, also affecting reported percentages. (–) indicates that percentages are not reportable to prevent back-calculation of potentially identifiable data.

a ‘Other’ RTs included: ‘stereotactic RT’; ‘intensity modulated RT, image guided RT’; ‘individual conformal RT’; and ‘individual conformal RT, image guided RT’

IQR: interquartile range; MPM: malignant pleural mesothelioma; RT, radiotherapy; SACT: systematic anticancer therapy.

More than half of the overall patient population had no record of one of the prespecified treatment modalities within 180 days of MPM diagnosis (52%; *n =* 455) (Supplementary Table S4). The median age at MPM diagnosis was 69.2 years (IQR, 63.8–75.2) for those with a treatment record and 74.1 years (IQR, 67.9–79.4) for those without (Supplementary Tables S4 and S5). The distribution of patients by histology was similar for the treated and untreated groups; both had a higher proportion of patients with non-epithelioid histology (69% [*n* = 290] and 67% [*n =* 305], respectively) (Supplementary Tables S4 and S5). In total, 32% (*n* = 150) of patients with no record of treatment died within 180 days versus 14% (*n* = 60) of the patients with a record of treatment within 180 days (Supplementary Tables S4 and S5).

### Overall survival

**Figure 2:**
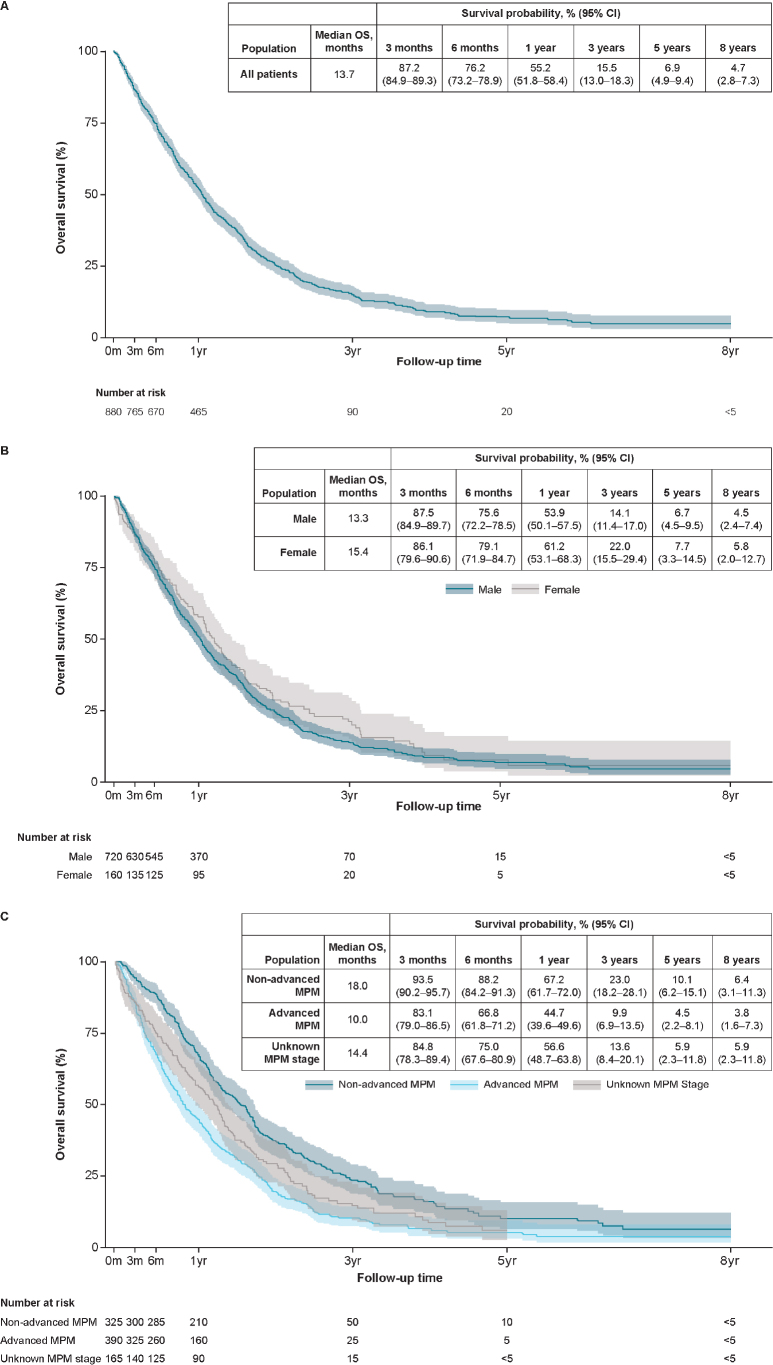
(A) Overall survival (overall patient population). (B) Overall survival by sex (overall patient population). (C) Overall survival by MPM stage. CI: confidence interval; MPM: malignant pleural mesothelioma; OS: overall survival; yr: year. Frequencies rounded to nearest 5.

In the overall patient population with MPM, median OS was 13.7 (95% confidence interval [CI], 12.6–14.6) months with survival probabilities of 55.2% (95% CI, 51.8–58.4) at 1 year and 6.9% (95% CI, 4.9–9.4) at 5 years (Figure 2A). Median OS was 15.4 (95% CI, 13.0–18.0) months in female patients and 13.3 (95% CI, 12.1–14.3) months in male patients (Figure 2B) and 10.0 (95% CI, 8.8–11.9) months in patients with advanced MPM. (Figure 2C). At 5 years, the survival probability was 10.1% in the non-advanced MPM group (95% CI, 6.2–15.1), 4.5% (95% CI, 2.2–8.1) in the advanced MPM group, and 5.9% (95% CI, 2.3–11.8) in the unknown MPM stage group (Figure 2C). Among patients receiving any surgery, median OS (95% CI) was 28.6 (25.3–31.6) months with survival probabilities of 92.3% (95% CI, 86.1–95.8) at 1 year and 17.9% (95% CI, 10.1–27.5) at 5 years. Median OS (95% CI) among patients who received any RT was 6.8 (6.0–7.8) months with survival probabilities of 18.8% (95% CI, 11.6–27.4) at 1 year and 1.8% (95% CI, 0.2–7.8) at 5 years. Among patients receiving any SACT, the median OS (95% CI) was 15.3 (13.9–17.6) months with survival probabilities of 63.8% (95% CI, 57.7–69.3) and 5.7% (95% CI, 1.8–12.7) at 1 and 5 years, respectively.

### Predictors of mortality in patients diagnosed with MPM compared with the general population

**Figure 3:**
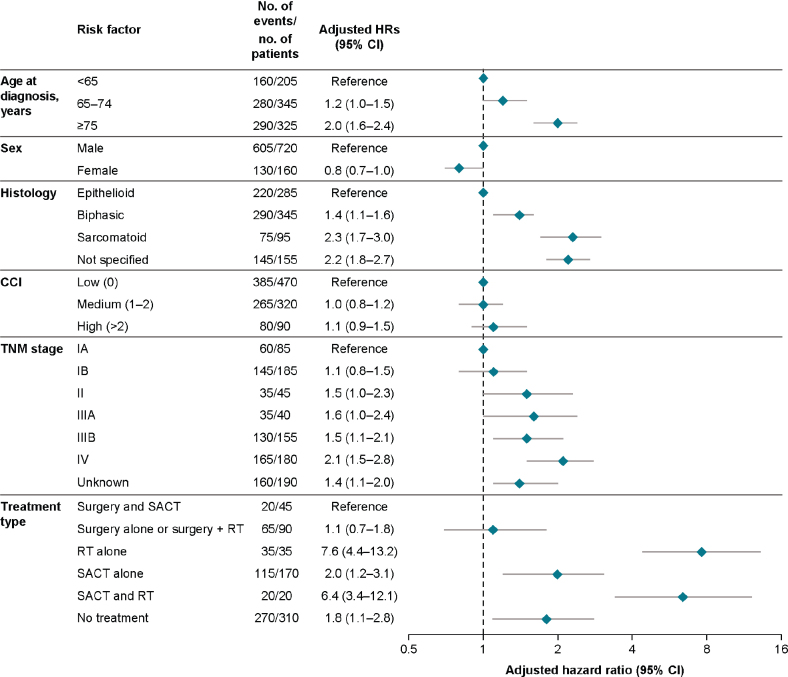
Predictors for overall survival up to 8 years. Hazard ratios obtained from Cox regression model with time in study as underlying time variable, adjusting for all other factors shown in the figure. CCI: Charlson Comorbidity Index; HR: hazard ratio; MPM: malignant pleural mesothelioma; RT: radiotherapy; SACT: systematic anticancer therapy; TNM: tumour, node, metastasis. Frequencies rounded to nearest 5.

Over up to 8 years of follow-up, the mortality rate was highest in patients with MPM aged 75 years and above at diagnosis compared with the reference group used for analysis of patients under 65 years (adjusted hazard ratio [aHR], 2.0 [95% CI, 1.6–2.4]) and in patients with sarcomatoid (aHR, 2.3 [95% CI, 1.7–3.0]) or unspecified histology (aHR, 2.2 [95% CI, 1.8–2.7]) compared with epithelioid histology (Figure 3). The presence of a medium or high CCI score compared with the reference (low CCI score) did not appear to affect mortality risk (Figure 3). Patients classified as TNM stage IV had a higher mortality risk (aHR, 2.1 [95% CI, 1.5–2.8]) than those classified at all other TNM stages based on patients with TNM stage IA as the reference (Figure 3).

## Discussion

In this study, based on routinely collected data from Danish registries between 2011 and 2018, the characteristics of the patient population were consistent with the profile of patients diagnosed with MPM [24, 34, 35], with many patients aged over 70 years and with an initial diagnosis of advanced MPM for most patients. MPM is challenging to diagnose, requiring radiological and preferably histological (mandatory for firm pathological diagnosis) investigations, each of which have limitations [12]. This may be reflected in the large proportion of patients in this study who were categorized with an unknown MPM stage. Almost half of the patients in this study had advanced MPM at diagnosis, and a further fifth of patients, although having a diagnosis of MPM, did not have a confirmed disease stage, making treatment decisions challenging. Pleural biopsy is considered to be the gold standard for confirmation of MPM [5], but given the typical age of patients with MPM, the invasive nature of biopsy can be suboptimal and the poor health of patients may mean that only palliative treatment is feasible [12, 24]. Radiological interpretation and staging are also difficult due to the heterogenous nature of MPM tumours [12] as reflected in the 22% of patients in this study who lacked TNM data.

The presence of sarcomatoid or biphasic histology is associated with a significantly worse outcome than epithelioid histology [36], with a median OS of 4 months, compared with a median OS of 13 months [12] when not treated with dual immunotherapy drugs. Therefore, it is crucial to identify histological status as early as possible to guide appropriate treatment decisions [24]. Here, histology was unknown for 18% of the patients, demonstrating a significant gap in available data upon which to base treatment decisions. In this study, the higher proportion of patients with non-epithelioid histology contrasts with previously published results. Baas et al. [24] reported only 7.3% of patients with biphasic histology, yet had similar results to those presented here for epithelioid and sarcomatoid histology; however, the large proportion of patients designated with ‘not otherwise specified’ histology (43%) in that study suggests that this may account for this difference.

The shorter median time-to-initial treatment seen here in patients with advanced MPM versus non-advanced or unknown MPM may reflect the urgency to treat based on the poor prognosis in patients with advanced MPM. The standard first-line therapeutic approach for MPM is surgery and chemotherapy with RT recommended in only a highly selected group of patients or as palliation, preventive treatment, or part of a multimodal treatment plan; however, most patients here were not eligible for surgical resection and as such palliative chemotherapy was the primary treatment option at the time of this study [37]. The low number of patients receiving RT (11%) as initial treatment compared with those receiving SACT or surgery (31% and 15%, respectively) in our study is consistent with these recommendations [3, 5]. In this study, SACT was the most frequently used initial therapy for all patients, as recommended in the published European guidelines at the time [3, 5]; however, the number of patients receiving SACT was still relatively low at 31% overall. Furthermore, only 11% and 7% of patients received cisplatin + pemetrexed or carboplatin + pemetrexed, respectively, despite these being the officially recommended treatment regimens. These results are consistent with those reported in other real-world studies and are likely to reflect the fact that many of these patients are elderly and/or frail and are either not referred to a centre of expertise for treatment, or do not want treatment due to the increased likelihood of comorbidities associated with older age [24]. The low number of patients with advanced MPM undergoing surgery is also reflective of the poor prognosis in this patient group, with many patients not eligible based on late diagnosis of the disease, and debate around the effectiveness of surgery [38]. Overall, a large proportion of patients with MPM were untreated with the treatment options categorized here (~50%), illustrating the lack of suitable therapeutic options available at the time of this study.

Our estimates for median OS are longer than the previously reported median OS of 12.5 months for men and 13.3 months for women in Denmark; however, those data were from a more narrowly defined time period (2008–2009) and may not reflect longer-term patterns [39]. Across the whole MPM population, median OS was longer in this study than reported figures in other European countries. In the UK, OS ranged from 8 months between 2013 and 2017 [24] to 9.5 months between 2007 and 2011 [34]. Median OS was also lower in Belgium (10.7 months) and the Netherlands (9.2 months) [34]. The somewhat better survival observed in our study may be due to several factors: the Danish Ministry of Health has provided specific guidance since 2004 on diagnosis and treatments for a range of malignancies (including MPM) in order to speed up diagnosis and treatment. MPM treatment is also centralized, with surgical evaluation restricted to one centre in Copenhagen, where a defined protocol using both neoadjuvant and adjuvant chemotherapy was used with surgery performed by only four surgeons. Finally, treatment for inoperable patients was limited to two centers, resulting in homogeneous treatment in the hands of very experienced physicians, which are factors known to improve clinical results. Nevertheless, the absolute difference in median OS between this and other studies was only a few months, and our study confirmed that a MPM diagnosis is associated with a very short median OS.

As expected, the mortality rate was higher in patients with advanced MPM compared with non-advanced MPM. The higher mortality risk associated with sarcomatoid histology is consistent with previously published data [16]; the higher risk associated with unspecified histology would suggest that a lack of accurate diagnosis for these patients limits the ability to accurately provide appropriate treatment to improve prognosis. Comorbidities did not appear to affect mortality risk based on CCI score; however, given the rapid progression of MPM and the overall poor outlook for patients, it is unlikely that any comorbidity would have a profound effect on disease progression. Mortality risk was higher in patients receiving RT; however, this is likely to correspond to the use of RT as palliative care.

Since the period of this study, the approval of nivolumab plus ipilimumab as first-line immunotherapy has provided great promise for patients with MPM. In the recent phase III CheckMate 743 trial, median OS was 18.1 months in patients receiving nivolumab plus ipilimumab compared with 14.1 months in the group receiving chemotherapy [19]. Nivolumab plus ipilimumab is now the recommended first-line therapy in the European Society for Medical Oncology guidelines [22]. Furthermore, other immunotherapy-based regimens are being investigated for first-line and previously treated MPM [40–42]. However, more time is required to assess the real-world impact of immunotherapy use in patients with MPM; therefore, establishing a treatment and outcomes baseline using available real-world evidence is necessary to accurately monitor changes in disease management and patient outcomes as these therapies become more widely used in clinical practice [24].

### Strengths and limitations

A key strength of this study was the use of nationwide population records that are based on mandatory tumour reporting using data that are linked by personal patient identity code across multiple registries. The study also included all patients with MPM over an 8-year period and included data on stage and histology at diagnosis and hospital comorbidity. In terms of limitations, the algorithm used to define initial treatment in this study was not validated in the Danish setting, and therefore there may be misclassification of the initial treatment status due to unknown completeness of SACT records in the Danish National Registry of Patients. Moreover, the completeness of the recorded therapy has not been assessed, so underestimation of the proportion of the treated patients cannot be ruled out. There were also no data on occupation and smoking status, and there was a high proportion of missing data on stage and histology. Additionally, Eastern Cooperative Oncology Group performance status, a key variable for treatment decision-making, was not recorded in the available data sources. Finally, biphasic mesothelioma is not classified as advanced MPM according to the current stage classification; however, in this study, biphasic MPM is included in the advanced prognosis group due to the poor outcome for patients with this type of histology. For this reason, a small group of patients with biphasic histology and stage I–IIIA disease, who were considered to be suitable for surgery may have been recorded in patient groups that do not strictly match the defined treatment groups of ‘non-advanced MPM’, ‘advanced MPM’, and ‘unknown MPM stage’. The inclusion of these patients in these groups may alter the overall OS results for that group due to the inclusion of patients with a better or a worse prognosis than the rest of the group that they have been assigned to.

## Conclusions

The results from this study provide information on the patient characteristics, initial treatment patterns, estimates of OS, and predictors of mortality in patients with MPM in Denmark between 2011 and 2018 and highlight the challenges associated with diagnosis and treatment of this rare and aggressive form of malignancy. A diagnosis of MPM was associated with high mortality among all patients and was consistent with data reported from other European countries. These results provide a baseline upon which to evaluate impact of newer treatments on survival and treatment outcomes in patients with MPM as these therapies become available for routine clinical practice.

## Supplementary Material

Patient characteristics, treatment patterns, and survival outcomes for patients with malignant pleural mesothelioma in Denmark between 2011 and 2018: a nationwide population-based cohort study

## Data Availability

The data from this study are not publicly available, and no individual data sharing is allowed.
