# Supplementary material for: Patient characteristics, treatment patterns, and survival outcomes for patients with malignant pleural mesothelioma in Denmark between 2011 and 2018: a nationwide population-based cohort study
Source: Acta Oncol. 2024 Aug 8;63:34802. doi: 10.2340/1651-226X.2024.34802 (PMC11332510; doi:10.2340/1651-226X.2024.34802)
Supplement: Patient characteristics, treatment patterns, and survival outcomes for patients with malignant pleural mesothelioma in Denmark between 2011 and 2018: a nationwide population-based cohort study [file AO-63-34802-s1.pdf]

Supplementary material has been published as submitted. It has not been copyedited, or typeset by Acta Oncologica

Supplementary Information: Table of Contents

Supplementary Tables .... **Fel! Bokmärket är inte definierat.**

Supplementary Methodology Tables 2

Supplementary Table S1. Variables used to define demographic and clinical characteristics 2

Supplementary Table S2. Variables used to define treatment patterns 3

Supplementary Results Tables.. 4

Supplementary Table S3. Initial treatment therapy combinations for patients with MPM. . 4

Supplementary Table S4. Clinical characteristics of patients with MPM not treated within 180 days after MPM diagnosis, both overall and by patient group. 5

Supplementary Table S5. Clinical characteristics of patients with MPM treated within 180 days after MPM diagnosis, both overall and by patient group. 7

Supplementary Figure 9

Supplementary Figure S1. Definition of study cohorts 9

Supplementary Methodology Tables

Supplementary Table S1. Variables used to define demographic and clinical characteristics

**Variable**	**Definition**
Diagnosis year	In categories or by year
Age at diagnosis (Years)	Continuous<br>• Mean (standard deviation)<br>• Median (interquartile range)<br>• Categories (<65, 65–75, >75)
Calendar year of diagnosis MPM	2011–2018 by year or in clinically/diagnostically relevant period
Sex	• Male<br>• Female
Other malignancy	Any available history of another primary malignancy (Yes/No)
Comorbidity profile	Charlson Comorbidity Index (CCI) score [1]<br>Comorbidities will be reported by the CCI, which will be calculated based on comorbidities based on diagnoses in the Danish National Patient Registry. A baseline CCI score will be calculated at index date, where each comorbidity has a specific weight and may be categorized into 3 levels (index score in parenthesis): low (0), medium (1–2) and high (>2) giving patients a value from 0 to 17 where 0 indicates no comorbidities and 17 indicates all comorbidities. Individual CCI comorbidities will be reported.
	Comorbid conditions: yes/no (if known)<br>• Parenchymal asbestosis<br>• Pleural abnormalities<br>• Other
Disease stage	• TNM staging will be used to define disease stage where available<br>• TNM stage as well as the detail of the T, N and M combination will be provided
Disease status at diagnosis	• Non-advanced MPM<br>• Advanced MPM<br>• Unknown MPM stage
Metastasis	• Metastasis at diagnosis date (Yes/No)<br>• Number of metastatic organ sites at index date (1,2,3) Location of metastases (at time of diagnosis and at time of regimen start):<br>- Non-visceral (Yes/No)<br>o Lymph nodes (Yes/No)<br>o Skin and soft tissue (Yes/No)<br>o Bone (Yes/No)<br>- CNS (brain and spinal cord) (Yes/No)<br>- Brain (Yes/No)<br>- Visceral<br>o Liver (Yes/No)<br>o Lung (Yes/No)<br>o All other locations (Yes/No)

1. Charlson ME, Pompei P, Ales KL, et al. A new method of classifying prognostic comorbidity in longitudinal studies: development and validation. J Chronic Dis. 1987;40(5):373-383.

CCI, Charlson Comorbidity Index; CNS, central nervous system; MPM, malignant pleural mesothelioma; RT, radiotherapy; SACT, systematic anti-cancer therapy; TNM, tumour, node, metastasis.

Supplementary Table S2. Variables used to define treatment patterns

Variable	Definition
Treatment modality during the 180 days following initial MPM diagnosis	Treatment modalities were described using the following categories (not mutually exclusive):<br>• Any surgery (Yes/No) (all combinations at any time during follow-up)<br>• Any SACT treatment (Yes/No) (all combinations at any time during follow-up)<br>• Any RT (Yes/No) (all combinations at any time during follow-up)
Initial treatment during 180 days following initial MPM diagnosis	Categories including combination therapies mutually exclusive:<br>• Any surgery (as initial treatment) (Yes/No)<br>• Any RT (as initial treatment) (Yes/No)<br>• Any SACT (as initial treatment) (Yes/No)
Surgery type (Surgery with radical intent only) during 180 days following initial MPM diagnosis	Variable categories:<br>• Decortication of pleura<br>• Unspecified open excision of pleura<br>• Open excision of lesion of pleura<br>• Other specified open excision of pleura<br>• Total pneumonectomy
Initial treatment categories during 180 days following initial MPM diagnosis	For incident MPM patients, the current study described the initial treatment category, which is the treatment and regimens started during 180 days after the initial MPM diagnosis.<br><br>Initial treatment is defined as the first treatment following diagnosis.<br>It was defined using the following categories:<br>• Surgery and SACT<br>• Surgery and RT and SACT<br>• Surgery alone or surgery and RT<br>• RT alone<br>• SACT alone<br>• SACT and RT<br>• No treatment (no surgery and no RT and no SACT)
First regimen following initial treatment during 180 days following initial MPM diagnosis	For all patients (including non-advanced, advanced and unknown MPM stage), the SACT regimen received at any time during initial treatment was described.
Time from diagnosis to treatment initiation	Time from diagnosis until date of initiation of the treatment:<br>Continuous (mean with SD, median with quartiles)<br>• 0–45 days<br>• 46–90 days<br>• 91–180 days<br>• >180 days

MPM, malignant pleural mesothelioma; RT, radiotherapy; SACT, systematic anti-cancer therapy.

Supplementary Results Tables

Supplementary Table S3. Initial treatment therapy combinations for patients with MPM.

	**All (*N*=880)**	**Non-advanced MPM (*n*=325)**	**Advanced MPM (*n*=390)**	**Unknown stage MPM (*n*=165)**
**Therapy combination, n (%)**				
Surgery and SACT (and no RT)	45 (5)	35 (11)	5 (–)	5 (–)
Surgery alone or surgery + RT (no SACT)	90 (10)	45 (15)	25 (6)	15 (10)
RT alone	60 (7)	15 (4)	35 (9)	10 (7)
SACT alone	195 (22)	65 (21)	90 (24)	35 (21)
SACT and RT (no surgery)	35 (4)	10 (3)	20 (5)	5 (–)
No treatment	455 (52)	150 (46)	215 (54)	95 (57)

MPM, malignant pleural mesothelioma; RT, radiotherapy; SACT, systematic anti-cancer therapy.

Supplementary Table S4. Clinical characteristics of patients with MPM not treated within 180 days after MPM diagnosis, both overall and by patient group.

	All (*N*=455)	Non-advanced MPM (*n*=150)	Advanced MPM (*n*=215)	Unknown MPM stage (*n*=95)
**Year of diagnosis, n (%)**				
2011	75 (16)	30 (20)	40 (18)	5 (–)
2012	80 (18)	35 (22)	45 (20)	5 (–)
2013	65 (14)	25 (16)	30 (13)	15 (15)
2014	65 (15)	20 (12)	30 (14)	20 (22)
2015	75 (17)	25 (17)	35 (17)	15 (17)
2016	45 (10)	10 (7)	20 (9)	15 (18)
2017	30 (7)	5 (–)	15 (–)	10 (13)
2018	10 (3)	5 (–)	5 (–)	0 (>0)
**Male, n (%)**	375 (82)	125 (83)	175 (82)	75 (82)
**Age group, n (%)**				
<65	80 (18)	25 (15)	45 (21)	15 (14)
65–74	160 (35)	60 (41)	70 (33)	30 (31)
≥75	215 (47)	65 (44)	100 (46)	50 (55)
**Age at MPM diagnosis, years**				
Median	74.1	73.4	74.0	75.8
IQR	67.9–79.4	68.1–78.4	67.2–79.2	71.0–81.0
**Died within 180 days, n (%)**	150 (32)	30 (20)	85 (40)	35 (35)
**Follow-up time, months**				
Median	10.9	13.9	8.2	12.2
IQR	4.0–21.7	7.1–27.2	3.1–19.0	3.4–23.5
**CCI, n (%)**				
Low (0)	235 (52)	90 (59)	100 (47)	45 (51)
Medium (1–2)	160 (36)	45 (29)	85 (41)	30 (34)
High (>2)	60 (13)	20 (13)	25 (12)	15 (15)
**Histology, n (%)**				
Biphasic	170 (37)	60 (39)	80 (38)	30 (34)

	All (*N*=455)	Non-advanced MPM (*n*=150)	Advanced MPM (*n*=215)	Unknown MPM stage (*n*=95)
Epithelioid	155 (34)	70 (47)	55 (26)	30 (31)
Sarcomatoid	40 (9)	0	40 (18)	0
Not specified	95 (20)	20 (15)	40 (18)	30 (34)
**TNM stage, n (%)**				
I–II	135 (30)	125 (83)	10 (–)	0
III	110 (24)	25 (16)	85 (41)	0
IV	100 (22)	0	100 (47)	0
Unknown	105 (23)	0	10 (–)	95 (100)

All frequencies rounded to nearest 5, also affecting reported percentages. (–) indicates that percentages are not reportable to prevent back-calculation of potentially identifiable data

CCI, Charlson Comorbidity Index; IQR, interquartile range; MPM, malignant pleural mesothelioma; TNM, tumour, node, metastasis; Q, quartile.

Supplementary Table S5. Clinical characteristics of patients with MPM treated within 180 days after MPM diagnosis, both overall and by patient group.

	All (*N*=420)	Non-advanced MPM (*n*=175)	Advanced MPM (*n*=180)	Unknown MPM (*n*=70)
**Year of diagnosis, n (%)**				
2011	40 (9)	20 (12)	15 (9)	5 (–)
2012	40 (9)	10 (6)	20 (12)	10 (11)
2013	40 (9)	20 (10)	15 (8)	5 (–)
2014	50 (12)	20 (11)	25 (13)	10 (13)
2015	55 (13)	25 (14)	20 (11)	10 (13)
2016	65 (16)	30 (17)	25 (14)	10 (17)
2017	95 (23)	35 (21)	40 (22)	20 (30)
2018	35 (9)	15 (9)	20 (11)	5 (–)
**Male, n (%)**	345 (82)	135 (79)	150 (83)	60 (85)
**Age group, n (%)**				
<65	125 (30)	60 (34)	45 (26)	20 (31)
65–74	185 (44)	85 (49)	80 (44)	25 (32)
≥75	110 (26)	30 (17)	55 (30)	25 (37)
**Age at MPM diagnosis, years**				
Median	69.2	68.3	69.8	70.5
IQR	63.8–75.2	63.4–73.6	64.4–76.3	63.2–77.4
**Died within 180 days, n (%)**	60 (14)	10 (5)	45 (25)	10 (11)
**Follow-up time, months**				
Median	13.9	17.3	10.5	15.1
IQR	8.1–22.1	11.3–27.8	6.0–18.3	9.3–21.7
**CCI, n (%)**				
Low (0)	235 (55)	90 (52)	105 (59)	40 (55)
Medium (1–2)	155 (37)	65 (38)	60 (35)	30 (–)
High (>2)	30 (8)	15 (10)	10 (6)	5 (–)
**Histology, n (%)**				
Biphasic	175 (42)	95 (54)	50 (27)	35 (51)

	All (*N*=420)	Non-advanced MPM (*n*=175)	Advanced MPM (*n*=180)	Unknown MPM (*n*=70)
Epithelioid	130 (31)	55 (32)	50 (29)	25 (32)
Sarcomatoid	55 (13)	0	55 (30)	0
Not specified	60 (15)	25 (14)	25 (14)	10 (17)
**TNM stage, n (%)**				
I–II	175 (42)	160 (91)	15 (–)	0
III	85 (20)	15 (9)	70 (38)	0
IV	80 (19)	0	80 (44)	0
Unknown	85 (20)	0	15 (–)	70 (100)

All frequencies rounded to nearest 5, also affecting reported percentages. (–) indicates that percentages are not reportable to prevent back-calculation of potentially identifiable data

CCI, Charlson Comorbidity Index; IQR, interquartile range; MPM, malignant pleural mesothelioma; TNM, tumour, node, metastasis.

Supplementary Figure

Supplementary Figure S1. Definition of study cohorts*

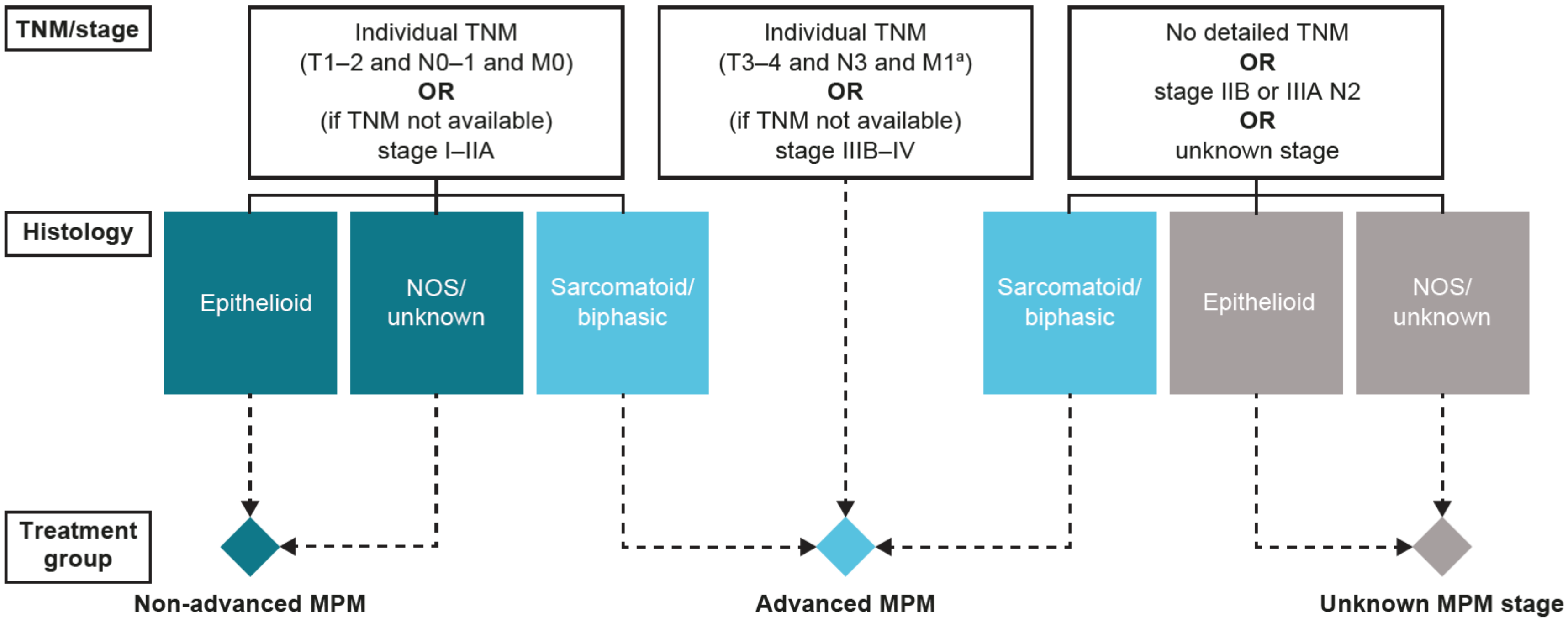

[a]Or visceral metastases -180/+180 days from MPM diagnosis.

* In a small number of cases, patients with biphasic histology and stage I–IIIA disease, who were considered to be suitable for surgery may have been recorded in patient groups that don’t strictly match the defined treatment groups defined here.

MPM, malignant pleural mesothelioma; NOS, not otherwise specified; TNM, tumour, node, metastasis.
